# Preferences of support and barriers and facilitators to help-seeking in pregnant women with severe fear of childbirth in Sweden: a mixed-method study

**DOI:** 10.1186/s12884-024-06580-2

**Published:** 2024-05-25

**Authors:** Carita Nordin-Remberger, Michael B. Wells, Joanne Woodford, Karin S. Lindelöf, Margareta Johansson

**Affiliations:** 1 Obstetric and Reproductive Health Research, Department of Women´s and Children´s Health, Uppsala University, Uppsala, 752 37 Sweden; 2 Women’s Mental Health during the Reproductive Lifespan – WOMHER, Uppsala University, Uppsala, Sweden; 3 Women’s and Children’s Health, Karolinska Institute, Solna, Sweden; 4 Healthcare Sciences and e-Health, Department of Women´s and Children´s Health, Uppsala University, Uppsala, Sweden; 5 Centre for Gender Research, Uppsala University, Uppsala, Sweden

**Keywords:** Barriers, Counselling, Facilitators, Fear of childbirth, FOBS, Mixed-method, Pregnancy, Support preferences, Women

## Abstract

**Background:**

There are few support interventions for women with fear of childbirth tailored towards type of fears and parity. To inform the future development of an acceptable and relevant intervention for women with severe fear of childbirth, primary objectives were to examine: (1) pregnant women’s experiences of and preferences for support and (2) barriers and facilitators to help-seeking. Secondary objectives were to examine if there are any differences based on pregnant women’s parity.

**Methods:**

Pregnant women with a severe fear of childbirth in Sweden completed an online cross-sectional survey between February and September 2022. Severe fear of childbirth was measured using the fear of childbirth scale. Quantitative data were analysed using descriptive and inferential statistics and free answers were analysed using manifest content analysis. A contiguous approach to integration was adopted with qualitative and quantitative findings reported separately.

**Results:**

In total, 609 participants, 364 nulliparous and 245 parous women, had severe fear of childbirth. The main category “A twisting road to walk towards receiving support for fear of childbirth” was explored and described by the generic categories: *Longing for support*, *Struggling to ask for support*, and *Facilitating aspects of seeking support*. Over half (63.5%), of pregnant women without planned or ongoing treatment, wanted support for fear of childbirth. Most (60.2%) pregnant women with ongoing or completed fear of childbirth treatment regarded the treatment as less helpful or not at all helpful. If fear of childbirth treatment was not planned, 35.8% of women would have liked to have received treatment. Barriers to help seeking included stigma surrounding fear of childbirth, previous negative experiences with healthcare contacts, fear of not being believed, fear of not being listened to, and discomfort of having to face their fears. Facilitators to help seeking included receiving respectful professional support that was easily available, flexible, and close to home.

**Conclusions:**

Most pregnant women with severe fear of childbirth felt unsupported during pregnancy. Findings emphasise the need to develop individual and easily accessible psychological support for women with severe fear of childbirth, delivered by trained professionals with an empathetic and respectful attitude.

**Supplementary Information:**

The online version contains supplementary material available at 10.1186/s12884-024-06580-2.

## Background

Fear of childbirth exists on a continuum, ranging from mild to severe and disabling [1–3]. When fear of childbirth during pregnancy is left untreated, there is an increased risk for pregnancy-related anxiety, which is further associated with impaired neuro-emotional development in newborns [4, 5]. High levels of anxiety during pregnancy may also interfere with a woman’s ability to cope with everyday life and preparations to become a mother [6]. Severe fear of childbirth can lead to avoidance of pregnancy, and is more prominent among foreign born woman living in Sweden [7]. Fear of childbirth is a common reasons for requesting a caesarean section (CS) [1–3], and associated with an increased use of epidurals during labour [8, 9], having a negative birth experience [10–13], and postpartum psychological ill-health [12, 14], that can negatively impact the mother-child relationship [15].

Most pregnant women prefer their partner to be present during childbirth [16] with their involvement contributing to positive outcomes for the mother [17], with women feeling empowered by having a supportive partner [18]. In Sweden, 98% of prospective fathers participate during the birth [19]. However, lack of partner support [18, 20], partner violence, and women’s dissatisfaction with the partnership [8] are associated an increased risk of fear of childbirth.

Despite these adverse impacts, fear of childbirth is an underrecognised and underprioritised healthcare issue due to factors such as lack of consensus of the definition of fear of childbirth [6], lack of structured treatments [21], and lack of routine screening during pregnancy [11, 22]. Psychological interventions including psychoeducation [PE] [23], cognitive behavioural therapy [CBT], enhanced midwifery care [24], psychodynamic therapy [PDT], and eye movement desensitization and reprocessing therapy [EMDR] [25–27], have been found to reduce fear of childbirth in pregnant women. However, the evidence base has been criticised due to poor methodological quality [24], and primarily focusing only on depression as an outcome [28]. Further, research suggests a need to develop fear of birth interventions that are better tailored to women’s individual needs [18]. Additionally, the majority of studies have not focused on pregnant women experiencing severe fear of childbirth [26, 29]; thus, there is a need for more research examining the experiences of and preferences for support in this population.

### Fear of childbirth in relation to parity

Both nulliparous and parous pregnant women may experience fear of childbirth [30]. Research suggests fear of childbirth in nulliparous women may stem from general fears starting in adolescence or early adulthood or prior history of anxiety disorders [31], whereas parous women with a previous traumatic birth experience are more likely to experience fear of childbirth during their next pregnancy [32]. Nulliparous women and parous women with a severe fear of childbirth may have different experiences and need different types of psychological support [12].

### The Swedish context for supportive counselling

In Sweden, approximately 110 000 women give birth annually [33], where 20% of expectant mothers suffer from severe fear of childbirth [34]. In 2020, approximately 10% of all pregnant women in Sweden received supportive counselling related to fear of childbirth [12]. Supportive counselling is delivered by a midwife, sometimes in cooperation with an obstetrician, psychologist, counsellor, psychiatrist, or behavioural therapist [35]. Supportive counselling has been found to help women feel safe and increase confidence in giving birth, and is related to a positive birth experience [9, 36]. Standard referral procedures for supportive counselling vary between Swedish antenatal care units. Midwives may ask women a general question about fear or use a screening instrument to identify fear of childbirth. Whilst some antenatal care units follow local referral guidelines, others require women to self-refer to a counselling unit [9]. Despite all antenatal clinics in Sweden offering supportive counselling for women with fear of childbirth [3], the structure and content of the counselling and its organisation differs greatly between clinics [3, 9].

### Research aim and objectives

The overall aim was to explore pregnant women with severe fear of childbirth and their preferences for support to inform the future development of a psychological intervention for severe fear of childbirth. primary objectives were to examine: (1) pregnant women’s experiences of and preferences for support and (2) barriers and facilitators to help-seeking. Secondary objectives were to examine if there are any differences in experiences of and preferences for support and barriers and facilitators based on pregnant women’s parity.

## Methods

### Design

A cross-sectional concurrent mixed-methods design [37], using an anonymous, online survey hosted by REDCap (Research Electronic Data Capture) [38], following the Checklist for Reporting Results of Internet E-Surveys [39]. Ethical approval was obtained from the regional ethical board in Sweden (Nr: 2021–03759.)

### Participants

Eligible participants were (1) 18 years of age or older; (2) self-identified as a pregnant woman, either nulliparous (i.e. a woman who has not given birth to a child) or parous (i.e. a woman who has given birth to any number of children); (3) living in Sweden; (4) able to read and understand Swedish or English enough to complete an online survey; and (5) had fear of childbirth (≥ 60 on the Fear of Birth Scale.

### Recruitment

A convenience sample was recruited from February 2022 to September 2022 via a number of recruitment strategies:

#### Hospital recruitment

Six hospitals across five counties in Sweden (Malmö, Karlstad, Stockholm, Uppsala, and Umeå) supported recruitment. Potential participants were informed about the study by a midwife either at the end of an appointment in the counselling clinic or at the routine ultrasound. Potential participants were handed a study information brochure with a survey weblink and QR (Quick Response) code for the survey (Appendix I).

#### Social medical advertisements

Advertisements were posted on social media (i.e., Facebook and Instagram), on websites, and in newsletters of non-profit organisations for pregnant women, groups for men and parents, and paid Facebook advertisements via an advertising company in collaboration with Uppsala University. Paid Facebook advertisements were used for approximately four weeks (July-August 2022). Study advertisement examples can be found in Appendix II. A survey web link and/or QR code was provided at the bottom of the advertisement.

### Procedure

#### Informed consent

Potential participants were informed about the study purpose via study information presented on REDCap. Study information stated participation was voluntary and would not affect their clinical care, and answers could be anonymous. Participants were advised that by clicking “yes” they consented to participate.

#### Survey

The survey comprised of three sections (1) Sociodemographic and obstetric characteristics including the Fear of Birth Scale (15 items) [15] (2) preferences for and experiences of support (9 items) [18] and (3) barriers and facilitators to help-seeking (9 items) [40] (Appendix III). Participants were able to review and/or change answers by using a back button. Survey functionality was tested by the research team. IP addresses were not stored in order to maintain participant anonymity.

#### Fear of birth scale

The Fear of Birth Scale is a self-report scale consisting of two 100 mm Visual Analogue Scales that are summed and then averaged. Participants answer the question “How do you feel right now about the approaching birth?” and by placing a mark the two scales (scale one ranges from calm to worried, and scale two ranges from no fear to strong fear). The cut-off point ≥ 60 as severe fear of childbirth and < 60 as no, mild or moderate fear of childbirth was used in accordance with previous research [14, 15, 34, 41, 42].

#### Open questions

Three open questions with free-text responses were included [37]: (1) “If no treatment is planned, would you like to receive treatment/support for your fear of childbirth?” and “If yes, please state why?”; (2) “What obstacles have you experienced in seeking help for your fear of childbirth?” and (3) “What would make it easier to seek help for fear of childbirth?”

#### Pilot testing

Research team members pilot-tested the online survey. After small logic errors and item-wording changes were made, the survey was pilot tested with three pregnant women prior to data collection to ensure the survey was understandable and acceptable. Feedback was provided via email or phone, and only minor wording changes were suggested and to clarify some items.

### Data analysis

#### Quantitative

We created several dichotomous variables to facilitate cross-tabulation. Age was recoded into two groups (1) < 35 years and (2) ≥ 35 (defining advanced maternal age, and reported to be associated with various pregnancy complications) [43, 44]. Relationship status was recoded into two groups (1) Cohabiting with a partner (married or living with someone) and (2) Not cohabiting (single or with a partner but not living together). Education was recoded into two groups (1) primary or high school (9–13 years) and (2) at least some or more college/university. Country of birth was recoded as (1) Sweden or (2) Other. Partner support was recoded into two groups (1) more partner support (those who responded “to a very large extent” or “to a fairly large extent”) and (2) less partner support (those who responded “to a small extent” or “not at all”). To be considered parous, participants needed to state “yes” that they had given birth to a child previously. Gestational week was recoded into three levels (1) < 25 weeks, (2) 25–36 weeks, and (3) > 36 weeks.

Data analysis was performed in IBM SPSS Statistics for Windows 28.0 (IBM Corporation). Descriptive statistics were used to compute frequencies, percentages, mean, and Standard Deviation [SD]. Chi-square test and Fisher´s Exact Test were used to assess differences in demographics and other covariates based on participants with severe fear of childbirth. We then compared differences between nulliparous and parous pregnant women. Thresholds for significance were set at *p* < .05.

#### Qualitative data

**Fig. 1:**
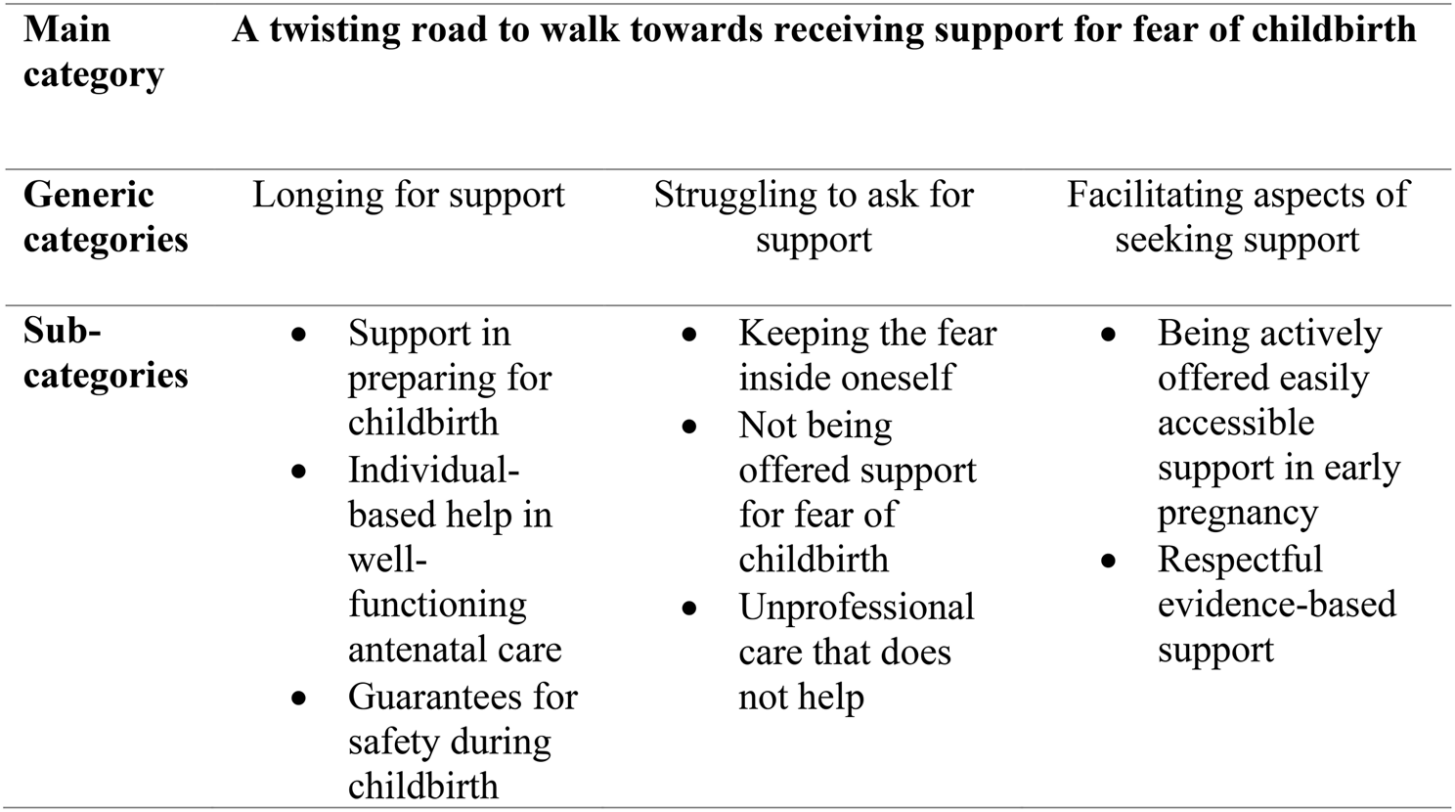
Findings of the qualitative data analysis

A manifest content analysis approach [45] was adopted to analyse responses to open survey questions informed by other mixed methods surveys [46]. Data analysis was managed using Word. Data about experiences of and preferences for support, barriers and facilitators to help-seeking were analysed separately. Three steps were followed to analyse the text (1) preparation, (2) organizing and (3) reporting. Two authors (CNR and MJ) read the free-text responses to gain an overall understanding of the free-text responses. Next, CNR and MJ identified condensed meaning units (e.g., text sharing a common meaning) and performed line-by-line coding separately. CNR reviewed initial meaning units and coding, with variations discussed by CNR and MJ. Subsequently, CNR and MJ sorted codes into three generic categories, including eight sub-categories that described the main category (Fig. 1). Identified categories were discussed by CNR and MJ to ensure mutual exclusivity and a credible foundation in data. Descriptions of main category, generic categories and sub-categories were prepared by CNR and MJ, with other co-authors performing peer examination of written descriptions. Trustworthiness was also established via independent coding by CNR and MJ and record keeping. To strengthen results, quotations from free text responses are presented, with quotations presented only with the women´s parity and age, to preserve confidentiality.

#### Data integration

A contiguous approach to data integration was adopted with qualitative and quantitative findings reported separately [47]. First, we analysed the quantitative data to describe the sample and examine experiences of and preferences for as well as barriers and facilitators to help-seeking. Second, we analysed the free-text responses to develop a deeper and more nuanced understanding of preferences for and experiences of support and barriers and facilitators to help-seeking.

## Results

### Participant characteristics

A total of 609 pregnant women were included: 60% (*n* = 364) were nulliparous and 40% (*n* = 245) were parous women. Participants had a mean age of 32.7 years (SD 4.305) and were, on average, in pregnancy week 26 (SD 9.734). Most participants were Swedish-born, lived with their partner, and had a university level of education (Table 1). Nulliparous women were more likely to be younger (*p* < .001), live in a city (*p* = .020), have experienced previous mental health difficulties (*p* = .024), and receive partner support (*p* < .001) compared to parous women. Parous women were more likely to be on parental leave (*p* = .008), and to have had previous experience of miscarriage (*p* < .001) compared to nulliparous women (Table 1).

**Table 1:** Sociodemographic and obstetric characteristics for pregnant women with severe fear of childbirth according to Fear of Birth Scale ≥ 60

Sociodemographic and obstetric characteristics	All women *n*=609 *n* (%)	Nulliparous women *n*=364 *n* (%)	Parous women *n*=245 *n* (%)	*p*-value
Age (Years)*				
<35	406 (66.7)	282 (78.1)	124 (51.2)	
≥35	197 (32.3)	79 (21.9)	118 (48.8)	<0.001
Civil status				
Cohabiting with partner	577 (94.7)	347 (95.3)	230 (93.9)	
Not Cohabiting	32 (5.3)	17 (4.7)	15 (6.1)	0.547
Highest level of education				
Primary or high school	125 (20.5)	71 (19.5)	54 (22.0)	
College/University	484 (79.5)	293 (80.5)	191 (78.0)	0.511
Employment status				
Employed	489 (80.3)	301 (82.7)	188 (76.7)	0.088
Studying	59 (9.7)	33 (9.1)	26 (10.6)	0.622
Parental leave	43 (7.1)	17 (4.7)	26 (10.6)	0.008
Sick leave	57 (9.4)	27 (7.4)	30 (12.2)	0.062
Sickness compensation and unable to work	5 (0.8)	4 (1.1)	1 (0.4)	0.653
Unemployed	14 (2.3)	10 (2.7)	4 (1.6)	0.422
Country of birth*				
Sweden	546 (89.8)	331 (91.2)	215 (87.8)	
Other	62 (10.2)	32 (8.8)	30 (12.2)	0.217
Place of residence				
City	426 (70.0)	270 (74.2)	156 (63.7)	
Town	99 (16.3)	52 (14.3)	47 (19.2)	
Village	84 (13.8)	42 (11.5)	42 (17.1)	0.020
Gestational week *				
<25	255 (41.9)	157 (43.3)	98 (40.3)	
25-36	249 (40.8)	149 (41.0)	100 (41.2)	
>36	102 (16.7)	57 (15.7)	45 (18.5)	0.612
Planned pregnancy				
Yes	342 (56.2)	200 (54.9)	142 (58.0)	
No	75 (12.3)	39 (10.7)	36 (14.7)	
Not the exact timing	192 (31.5)	125 (34.3)	67(27.3)	0.111
Status of abortion				
Yes	155 (25.5)	88 (24.2)	67 (27.3)	
No	454 (74.5)	276 (75.8)	178 (72.7)	0.432
Status of miscarriage				
Yes	150 (24.6)	69 (19.0)	81 (33.1)	
No	459 (75.4)	295 (81.0)	164 (66.9)	<0.001
Current mental health difficulties*				
Yes	229 (37.7)	147 (40.6)	82 (33.5)	
No	378 (62.3)	215 (59.4)	163 (66.5)	0.090
Previous mental health difficulties				
Yes	353 (58.0)	225 (61.8)	128 (52.2)	
No	256 (42.0)	139 (38.2)	117 (47.8)	0.024
Mode of birth preference if pregnancy medical uncomplicated*				
Vaginal	354 (58.3)	210 (58.0)	144 (58.8)	
Caesarean Section	138 (22.7)	84 (23.2)	54 (22.0)	
Do not know	115 (18.9)	68 (18.8)	47 (19.2)	0.945
Any experiences of partner violence*				
Yes	121 (19.9)	76 (21.0)	45 (18.4)	
No	486 (80.1)	286 (79.0)	200 (81.6)	0.489
Partner support				
To a very large extent	277 (45.5)	188 (51.6)	89 (36.3)	
To less extent	332 (54.5)	176 (48.4)	156 (63.7)	<0.001

* The number does not add up to 100% due to missing data

**Qualitative findings**

Qualitative analysis resulted in the main category “A twisting road to walk towards receiving support for fear of childbirth” described by three generic categories: (1) *Longing for support*, (2) *Struggling to ask for support*, and (3) *Facilitating aspects of seeking support* (Fig. 1).

### Experiences of and preferences for support

#### Quantitative findings

In total, 337 (63%) of pregnant women reported wanting to receive support in relation to fear of childbirth. A little over one-third (219/609, 36%) of pregnant women either planned to receive, currently received, or had completed fear of childbirth treatment. An additional one-third (36%) of pregnant women would like to receive treatment, but had not yet planned or started any treatment options (Table 2). Of those with ongoing or completed treatment, 60% (59/101) regarded the treatment as less helpful or not at all helpful. Treatment offered for fear of childbirth was described as mainly supportive counselling through clinical professionals (Table 2). Parous women were more likely to have had a planned treatment (*p* = .002), as well as want individual support (*p* = .019) compared to nulliparous women (Table 2).

**Table 2:** Experiences of and preferences for support

Experiences of and preferences for support	All women *n*=609 *n* (%)	Nulliparous women *n*=364 *n* (%)	Parous women *n*=245 *n* (%)	*p*-value
Would you like to receive support in relation to fear of childbirth? *				
Yes	337 (63.5)	200 (64.5)	137 (62.0)	
No	33 (6.2)	17 (5.5)	16 (7.2)	
No not know	161 (30.3)	93 (30)	68 (30.8)	0.672
Who would you like to deliver support for fear of childbirth? #				
Midwife	445 (73.1)	263 (72.3)	182 (74.3)	0.644
Psychologist	288 (47.3)	178 (48.9)	110 (44.9)	0.375
Physician	230 (37.8)	130 (35.7)	100 (40.8)	0.235
Counsellor	131 (21.5)	78 (21.4)	53 (21.6)	1.000
Have you received professional treatment for fear of childbirth?				
Treatment is planned	118 (20.2)	72 (20.7)	46 (19.4)	
On-going treatment	59 (10.1)	25 (7.2)	34 (14.3)	
Completed treatment	42 (7.2)	18 (5.2)	24 (10.1)	
No treatment is planned	365 (62.5)	232 (66.9)	133 (56.1)	0.002
What kind of treatment is completed, on-going or planned? *				
Counselling with midwife, physician, psychologist or counsellor	204 (98.1)	107 (97.3)	97 (99.0)	
CBT**, PDT*** or PE****	4 (1.9)	3 (2.7)	1 (1.0)	0.624
*If the treatment was ongoing or completed*				
To what extent do you think your professional treatment has helped you? *				
To a very large or large extent	39 (39.8)	16 (40.0)	23 (39.7)	
To a lesser extent or not at all	59 (60.2)	24 (60.0)	35 (60.3)	1.000
If no treatment for childbirth fear is planned, would you like to have it? *				
Yes	130 (35.8)	85 (37.0)	45 (33.8)	
No	67 (18.5)	36 (15.7)	31 (23.3)	
I do not know	166 (45.7)	109 (47.4)	57 (42.9)	0.194
How important is talking about your fear of childbirth? *				
It is very important	233 (38.2)	129 (39.3)	104 (45.6)	
It is important	237 (38.9)	149 (45.4)	88 (38.6)	
It is not important at all	86 (14.1)	50 (15.2)	36 (15.8)	0.251
Support preferences				
Individually for me	390 (64.0)	219 (60.2)	171 (69.8)	0.019
Together with my partner	416 (68.3)	258 (70.9)	158 (64.5)	0.116
In a group with other expectant mothers	142 (23.3)	91 (25.0)	51 (20.8)	0.272
In a group with other expectant mothers and fathers				
Talk to other parents	79 (12.9)	55 (15.1)	24 (9.8)	0.073.
Extra midwifery appointments	57 (9.3)	38 (10.4)	19 (7.8)	0.330
Antenatal parental course	233 (38.2)	139 (38.2)	94 (38.4)	1.000
Breathing and relaxation excercises	136 (22.3)	113 (31.0)	23 (9.4)	<0.001
The method of “Confident Birth”	158 (25.9)	109 (29.9)	49 (20.0)	0.008
Mindfulness	188 (30.8) 101 (16.5)	132 (36.3) 68 (18.7)	56 (22.9) 33 (13.5)	<0.001 0.113

# =Variables do not sum to 100% because of more than one professional support alternative were possible to choose

* The number does not add up to 100% due to missing data

**= Cognitive behavioural therapy

***=Psychodynamic therapy

**** =Psychoeducation

#### Qualitative findings

Sixty-seven women responded to the open question regarding their experiences of support and preferences for support. Analysis resulted in the generic category “Longing for support” and includes the sub-categories: *Support in preparing for childbirth, Individual-based help in well-functioning antenatal care*, and *Guarantees for safety during childbirth* (Fig. 1).

*Support in preparing for childbirth* concerns expressed preferences for receiving support to help prepare for childbirth. Nulliparous and parous women wanted to be informed about pain relief methods, how to reduce risk of infections in the uterus, and criteria for early hospital discharge after birth. Women expressed receiving information about the antenatal care organization and their routines could facilitate feelings of safety and instill a sense of security. To better prepare for childbirth, women wanted help to design a written birth plan in early pregnancy including wishes concerning mode of birth, length of hospital stay after the birth, and that the plan is respected, rather than questioned and negotiated by professionals. A parous woman aged 34 expressed her need for support:

*I would like to have a plan for how my birth will go and that the plan is also carried out to 100% to make me safer and reduce my fear of childbirth.*

Women wanted support from labour ward professionals and a doula, and to learn how to cope with their fears, seeing it as their right to seek and receive support.

Women perceived psychological support as something that could help them feel safer, reduce their fear of childbirth, and help to deal with anxiety experienced. A nulliparous woman aged 32 expressed a preference to *“Talk about it* [the birth] *with someone who takes me seriously. Probably need to process the last birth as well.”*

However, not all women who had received psychological treatment for fear of childbirth found the treatment helpful, with one parous woman, aged 30, describing her treatment experience as:

*Exposing me, bringing up my wounds, standing there alone with no support … a very naked vulnerable feeling. That the support* [offered] *is too insignificant in comparison to my own fear.*

*Individual-based help in well-functioning antenatal care* concerns women asking for well-organised antenatal care with adequate resources to allow pregnant women to attend a labour ward of their choosing, to process previous negative birth experiences, to receive continuous antenatal care during childbirth, and sufficient midwifery support during active labour. As one parous woman, aged 31, explained:

*I would like to talk about my previous birth, why it turned out the way it did, and talk through how the next birth can be different and what options I have and how I can prepare differently.*

Women expressed alternative antenatal care models, with being able to choose between several different types of care, to have a paid home birth with a midwife, and continuity of care, expressed as ways to feel supported. Receiving continuous support by a known midwife throughout antenatal, intrapartum, and postnatal care, and being assigned one specific midwife and physician during a hospital birth were also suggested.

*Guarantees for safety during childbirth* concerns women wanting to receive improved guarantees of safety and health and well-being of themselves and their baby during childbirth via increased ultrasound checks, receiving appropriate care, and that sufficient resources are available during childbirth. One parous woman, aged 39, expressed wanting to:

*Get guarantees that I will receive support and pain relief during labor and that the staff will minimize the risks for me, but above all, my baby, during labor.*

The receipt of pain relief during childbirth according to preference was also expressed as important, as were professionals working to minimize health risks for the woman and the baby as well as offering postnatal care follow-ups, especially after vaginal tears and caesarean section.

### Barriers to help seeking for childbirth fear

#### Quantitative findings

The most prevalent barriers for help-seeking were: fear of not being listened to by others, fear of not being believed by others, previous negative experience of health care contacts, and discomfort of having to face my own fears. Stigma surrounding fear of childbirth was a barrier for 14% of all pregnant women but higher for nulliparous women (*p* = .105). Parous women were more likely to regard previous negative experience of healthcare contacts as a barrier, compared to nulliparous women (*p* = .012) (Table 3).

**Table 3:** Barriers to help-seeking for childbirth fear

Barriers to help-seeking	All women *n*=609 *n* (%)	Nulliparous women *n*=364 *n* (%)	Parous women *n*=245 *n* (%)	*p*-value
Stigma surrounding fear of childbirth				
Yes	88 (14.4)	60 (16.5)	28 (11.4)	
No	521 (85.6)	304 (83.5)	217 (88.6)	0.105
Previous negative experience of healthcare contacts				
Yes	190 (31.2)	99 (27.2)	91 (37.1)	
No	419 (68.8)	265 (72.8)	154 (62.9)	0.012
Fear of not being believed in by others				
Yes	211 (34.6)	126 (34.6)	85 (34.7)	
No	398 (65.4)	238 (65.4)	160 (65.3)	1.000
Fear of not being listened to by others				
Yes	308 (50.6)	172 (47.3)	136 (55.5)	
No	301 (49.4)	192 (52.7)	109 (44.5)	0.055
Discomfort of having to face my own fears				
Yes	164 (26.9)	107 (29.4)	57 (23.3)	
No	445 (73.1)	257 (70.6)	188 (76.7)	0.114

#### Qualitative findings

Ninety-two women responded to the open question regarding barriers to help-seeking.

Analysis resulted in the generic category “Struggling to ask for support” and includes the sub-categories: *Keeping the fear inside oneself, Not being offered support for fear of childbirth* and *Unprofessional care that does not help* (Fig. 1).

*Keeping the fear inside oneself* concerns women expressing feeling *troublesome* and *awkward* about their encounters with antenatal care, experiencing difficulties explaining their fears to others and admitting their fear to themselves. Women struggled to explain what they were afraid of, leading to concerns that professionals would not believe them. Some women did not take their own fear seriously, believing others to have more to be afraid of to be in more need of more help. These women expressed not wanting to bother and burden professionals and hoped the fear would disappear by itself. Some women found it difficult to understand whether the fear was “normal” or whether they needed professional help. Women expressed feeling “*ridiculous”, “dumb”, “silly”*, and “*shameful*” and regarded their fear as just “*stupid thoughts”* and therefore there was no need to seek support. Women felt too tired, lacked energy, or were unmotivated to start dealing with their fear. A nulliparous woman, aged 35, explained:

*I don’t want more meetings as I don’t think there is much that can help, and I get so tired and exhausted from crying.*

Women also expressed *keeping the fear inside oneself* due to more practical concerns, for example, everyday life being time-consuming and needing to travel long distances to access help.

*Not being offered support for fear of childbirth* concerned women reporting not receiving support for their fear due to challenges contacting healthcare, difficulties understanding what support was available, and not knowing where to access help. Women described either no support being available or lack of availability of their preferred intervention. Women also expressed not being believed or taken seriously by professionals or their fear being dismissed as “*normal*” anxiety. One nulliparous aged 32 woman noted:

*The midwife at the antenatal clinic had instructions that you had to have a strong enough fear of childbirth to be referred to the Aurora clinic, I had to nag repeatedly which felt really hard”.*

Not receiving early support in pregnancy was experienced as particularly painful, and had a negative impact on mental health. One nulliparous aged 28 woman stated:

*I’m considering terminating the pregnancy because I don’t want to go through labour and my midwife said treatment for the fear only starts after the routine ultrasound.*

*Unprofessional care that does not help* concerns women not being offered professional or helpful care. Women expressed barriers such as lack of healthcare resources, limited appointments, long waiting times, and tired, stressed, irritated, and understaffed professionals. This led to women feeling not listened to and considering it pointless to continue to seek help. Some women considered antenatal care to be poorly organized, resulting in unhelpful care that was not worth seeking. For some women receiving support for their fear, support was perceived as unprofessional with professionals described as being “*uncomfortable”* in counselling support, “*ignorant”, “incompetent”, “sluggish”*, and *“unmotivated”* with a lack of understanding of their problems. One parous woman aged 33 stated:

*A midwife at the antenatal clinic who asked a ‘routine question’ about childbirth fear was surprised by the answer and my reaction. I felt like I needed to handle that* [the fear] *myself”.*

Some women perceived that the support offered would make no difference to either the women’s emotions of “*panic” and “loss of control”* or a negative birth outcome, with the fear considered hard to overcome. One nulliparous woman, aged 27, described it as: *“I couldn’t work the rest of the day after the counselling support and got a disturbed night’s sleep … the whole thing got even more traumatic”.* Another nulliparous woman, aged 33, said: “*I felt resignation when I received the support offered, I got the notion that there is nothing they can help me with”.*

Some women felt stigmatised by professionals, and perceived their fear to have been turned into a clinical diagnosis, creating a disadvantage in “*power*”, alongside misconceptions and preconceived notions on perinatal mental health and previous mental illness before childbirth. One nulliparous woman, aged 31, reported:

*My history with anorexia and anxiety often comes into focus.* [I have this] *feeling that everything comes down to making a diagnosis and it scares me. There have been errors in communication with the psychologist and I have received a diagnosis I do not feel comfortable with.*

### Facilitators to help-seeking for childbirth fear

#### Quantitative findings

Facilitators to help-seeking were receiving easily available help, professional support close to home, and being able to choose between different appointment times. There were no significant differences between nulliparous and parous women. It was regarded as important by women to have the opportunity to influence the care they received. For example, being able to choose between digitally-based meetings with lectures, and webinars was regarded as important. Digital solutions were considered to make it easier to seek help due to saving time on transportation and facilitating anonymity. Some women preferred to be offered physical meetings and to be allowed to visit the labour ward during pregnancy. Nulliparous women were more likely to prefer support by internet links (*p* = .007) and mobile applications (*p* = .009) compared to parous women (Table 4).

**Table 4:** Facilitators in seeking help for pregnant women with severe fear of childbirth according to Fear of Birth Scale ≥ 60

Facilitators in help seeking	All women *n*=609 *n* (%)	Nulliparous women *n*=364 *n* (%)	Parous women *n*=245 *n* (%)	*p*-value
Receiving professional support close to my home				
Yes	224 (36.8)	135 (37.1)	89 (36.3)	
No	385 (63.2)	229 (62.9)	156 (63.7)	0.916
Easily available help				
Yes	432 (70.9)	268 (73.6)	164 (66.9)	0.091
No	177 (29.1)	96 (26.4)	81 (33.1)	
Being able to choose between different times for appointments				
Yes	232 (38.1)	141 (38.7)	91 (37.1)	
No	377 (61.9)	223 (61.3)	154 (62.9)	0.755
Kind of support item preference				
Physical meetings	433 (71.1)	262 (72.0)	171 (69.8)	0.623
Internet links	196 (32.2)	133 (36.5)	63 (25.7)	0.007
Online meetings	155 (25.5)	90 (24.7)	65 (26.5)	0.684
Mobile applications	155 (25.5)	107 (29.4)	48 (19.6)	0.009
Leaflets	126 (20.7)	85 (23.4)	41 (16.7)	0.061
Telephone calls	74 (12.2)	36 (9.9)	38 (15.5)	0.051
Chatt	72 (11.8)	44 (12.1)	28 (11.4)	0.905
Short text messages	27 (4.4)	18 (4.9)	9 (3.7)	0.584

#### Qualitative findings

Ninety-four women responded to the open question regarding facilitators to seeking support. Analysis of responses resulted in the generic category “Facilitating aspects of seeking support” and includes the sub-categories: *Being actively offered easily accessible support in early pregnancy*, and *Respectful evidence-based support* (Fig. 1).

*Being actively offered easily accessible support in early pregnancy* concerns women describing being asked actively and directly by the midwife several times during pregnancy as a way of facilitating help-seeking. One nulliparous woman aged 34 wrote:

*The fact that the midwife asks about support for fear of childbirth is something I’m interested in. I’m afraid of feeling or appearing silly, immature and not ready to be a parent if I express fear of childbirth.*

Women considered being offered and encouraged to receive support before (e.g., for women with a previous traumatic birth experience) or in early pregnancy as ways to facilitate help-seeking. Women also wanted to be able to receive support for fear of childbirth without having to be referred for consultation. One parous woman aged 34 noted:

*If I had received the slightest help before I got pregnant, it would have felt easier to receive help during the pregnancy. I would have also had greater confidence that the maternity care genuinely wanted to help me based on my circumstances.*

*Respectful evidence-based support* concerns women expressing being offered respectful evidence-based support as a way to facilitate seeking help for their fear. Women wanted competent antenatal care professionals with knowledge and training in mental health, childbirth complications, and available support and treatment options. One nulliparous woman aged 30 described:

*Finally, I spoke to a psychologist who was also trained and had worked as a midwife for many years. I felt that she could guide me and to deal with my feelings. The support meant a lot to me and it gave me security.*

Women also expressed respectful care, for example, empathy, warmth, and understanding for women who are not within the “*norm*” as facilitating accessing support. Women wanted to be listened to, taken seriously, and not rejected, belittled, put down, ignored, trivialized, or questioned by professionals. One parous woman, aged 36, asked for: *“Professionals that have enough time to provide support, and offer suitable therapists who instill confidence”*.

It was expressed by women that having the right to decide over one’s own body and to choose the mode of birth was important also. Women did not want to receive coercive treatment and motivational talks for their fear with the aim of forcing women to give birth vaginally. One nulliparous woman aged 31 wrote: “[It] *must be easier to get a caesarian section in case of severe fear of childbirth.* [It is] *unreasonable to put patients through months of extreme anxiety”.*

## Discussion

Around two-thirds (63%) of women with fear of childbirth had not planned nor had received any supportive psychological treatment for fear of childbirth. Of those who had received support, 60% reported that the support received was less helpful or not helpful at all. Pregnant women preferred to receive individual-based support provided by antenatal healthcare professionals with sufficient mental health knowledge. Barriers to seeking support included a fear of not being listened to, or believed in by others, the discomfort of having to face their own fears, previous negative healthcare contacts, and the general stigma surrounding fear of childbirth. Facilitators to seeking support included respectful evidence-based support that was easily available, flexible, and close to home.

### Support for fear of childbirth

Swedish standard antenatal care states that women with fear of childbirth should be offered support via counselling with a midwife [21]. Our findings suggest not all pregnant women with severe fear of childbirth who had wanted psychological treatment actually received it. Some antenatal clinics may not support all pregnant women with a severe fear of childbirth, given the quality of clinical care and resources differs throughout Sweden [21].

Our findings indicate that parous women were more likely to receive a planned treatment for fear of childbirth than nulliparous women. This difference may partly be because parous women, especially those who had a complicated birth or negative birth experience, such as a caesarean section [48], are routinely invited to review their records with a physician or a midwife and offered a plan of care for the next birth [3]. A recent meta-analysis suggests nulliparous and parous women have similar levels of fear, but have fear for different reasons [12]; therefore, they may have different support needs. Qualitative findings suggested nulliparous women wanted support to prepare for childbirth via information and counselling with an individual birthing plan to help make them feel safe. This finding is in line with other research with nulliparous women, suggesting support may need to focus on handling the unknown and upcoming birth, receiving information on different childbirth scenarios, and injury prevention [12].

Importantly, 60% of the women who received support for fear of childbirth considered it less helpful or not helpful at all. Whilst Swedish research suggested women were satisfied with counselling, effects on fear, anxiety, and requests for a caesarean section have not been shown to differ from those not receiving supportive counselling [36]. Non-pharmacological interventions have been found to reduce fear of childbirth; however, effects were not clinically meaningful [18].

Importantly, 45% of pregnant women with severe fear of childbirth in the present study did not know if they wanted to receive psychological treatment. Previous research suggests pregnant women may believe they can manage their fear by not talking to others about it [18, 42, 49], and subsequently, refrain from participating in antenatal parental classes [49]. Parous women may adopt additional avoidant coping strategies such as blocking memories of previous traumatic childbirth experiences [49], and avoid discussing their upcoming birth with healthcare professionals [18].

### Barriers to help-seeking for childbirth fear

Barriers to help-seeking included fear of not being listened to, or believed in by others. Other research suggests women with fear of childbirth experience a lack of understanding from healthcare professionals [49], with their birthing wishes disregarded in favour for standardised procedures [50]. Pregnant women with fear of childbirth have described the importance of being treated by a competent midwife during counselling e.g., who is calm, skilled, whom acknowledges and listens to the women´s thoughts and feelings, thus enhancing a sense of trust and security [36]. However, our findings suggest previous negative healthcare contacts were a barrier to help-seeking. During pregnancy, women in Sweden have regular contact with an antenatal care midwife providing opportunities for the woman-midwife team to work with any previous negative healthcare experiences, and create new positive experiences [3]. Research has found parous women to be more likely to regard previous negative healthcare experiences as a barrier in seeking help for fear of childbirth, compared to nulliparous women [50].

Another barrier identified was stigma. Stigma has been found to be a barrier to the implementation of perinatal mental health policy and practice [24]. Feelings of isolation, guilt, and shame, due to perceived stigma have been reported by women with fear of childbirth in other research, with women feeling unable to talk about their fears with their partners or midwives since pregnancy is generally seen as a time of happiness [51]. Gendered norms and ideals about how a pregnant woman, or a woman in labour, should feel, think, and act, impact both the way healthcare professionals treat women and the way women express and behave themselves [52] and act as barriers to help-seeking.

### Facilitators in help-seeking for childbirth fear

Facilitators to help-seeking include the provision respectful evidence-based support that is easily available, flexible, and close to home. Examples of flexible solutions include digital self-help interventions [28, 53, 54] and healthcare professionals being able to provide support via telephone or video conversations, allowing care at a distance and facilitating access for women who have little time for physical visits e.g., full-time jobs and childcare responsibilities [28]. Interventions to overcome stigma for women with fear of childbirth may be important to develop, with such interventions found to be effective for reducing stigma in relation to perinatal depression [55] and may be helpful to enhance help-seeking in the population.

Results also suggest a need for midwives to be knowledgeable in psychological treatment for fear of childbirth, with women needing compassionate, respectful, and supportive care by healthcare professionals, regardless of fear and birthing preferences [49]. Facilitators to the effective implementation of perinatal mental health policy and practice include the provision of flexible women-centered services, delivered by knowledgeable healthcare professionals working for continuity of care [24]. The quality of the healthcare-provider relationship, healthcare professionals normalising perinatal mental health problems, knowing that help is available, and understanding different treatment options have been found to be facilitators to mental health screening among pregnant women [40].

Overall, results suggest a need for more research to develop and evaluate evidence-based psychological interventions for women with severe fear of childbirth that are tailored to women´s needs and preferences. Evidence suggests midwife-led continuity of care models with women supported throughout the childbirth period are beneficial for women with fear of childbirth [56, 57]. Policy makers need to consider Swedish systems of care and allow evidence-based care that optimises a woman´s chance of forming a trusting relationship with healthcare providers. However, the implementation of perinatal mental health policy and practice is complex and varied [24] and lack of training and knowledge of healthcare professionals, complex referral pathways, and lack of trusting relationships between the woman and healthcare providers have been found to be barriers to effective implementation [24].

### Strengths and the limitations

This study provides a comprehensive overview of the experiences of and preferences for support, barriers and facilitators to help-seeking, and it also examines differences based on pregnant women’s parity. Another strength is the relatively large sample size and that all counties in Sweden are represented. Despite these strengths, the study has several limitations. Recruitment relied on convenience sampling, and based on sample characteristics, our sample only represents Swedish-speaking women. Thus, findings may not be generalizable to non-Swedish-speaking women in Sweden. Due to the online survey format women needed to have some level of digital literacy to complete the survey which may have impacted the transferability of findings. Qualitative data consisted of free-text responses that varied in length and depth. However, the data still provided important insights into the experiences of women with severe fear of childbirth, their needs, and perceived barriers and facilitators to help-seeking.

## Conclusions

This is among the first studies in Sweden to provide a comprehensive overview of the experiences of and preferences for support of pregnant women with severe fear of childbirth and barriers and facilitators to help-seeking, and to examine differences based on pregnant women’s parity. Results indicate not all women with severe fear of childbirth receive support, and many who do receive support regard it as unhelpful. Developing a more comprehensive understanding of experiences and preferences for support, alongside barriers and facilitating for help-seeking, including taking into consideration differences based on women´s parity, are important first steps to inform the development of more acceptable,, relevant, and effective support for women with severe fear of childbirth. Findings highlight the importance of developing accessible, flexible, and individualised support, delivered by trained professionals with an empathetic and respectful attitude, enabling pregnant women to sustain their autonomy during pregnancy and childbirth.

### Electronic supplementary material

Below is the link to the electronic supplementary material.

Supplementary Material 1

Supplementary Material 2

Supplementary Material 3

## Data Availability

The datasets used and/or analysed during the current study are available on request from Carita Nordin-Remberger, Department of Women´s and Children´s Health, Uppsala University. Dag Hammarskjölds väg 20, 752 37 Uppsala, Sweden.

## Abbreviations

CBT: Cognitive behavioural therapy
CS: Caesarean Section
EMDR: Eye Movement Desensitization and Reprocessing
MRC: Medical Research Council
PE: Psychoeducation
PDT: Psychodynamic therapy
PT: Psychotherapy
SD: Standard Deviation
