# Supplementary figures and images for: Preferences of support and barriers and facilitators to help-seeking in pregnant women with severe fear of childbirth in Sweden: a mixed-method study
Source: BMC Pregnancy Childbirth. 2024 May 25;24:388. doi: 10.1186/s12884-024-06580-2 (PMC11127315; doi:10.1186/s12884-024-06580-2)

**APPENDIX I**

**Advertisment for hospital recruitment and social media.**

**
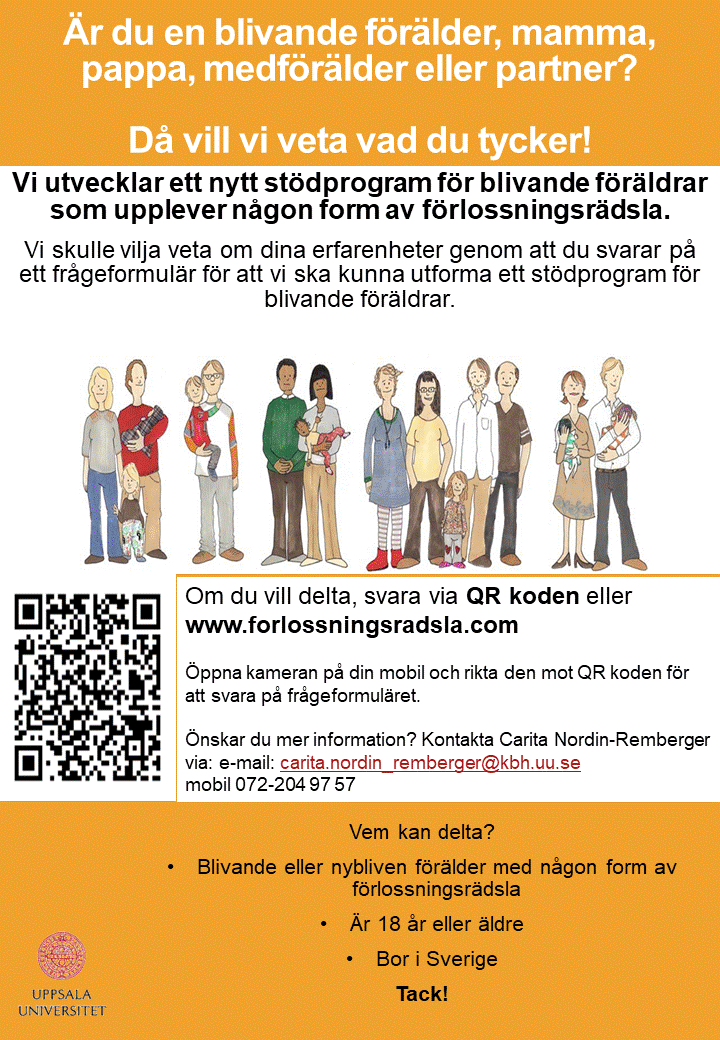
**

Supplement: Supplementary file 1 — Supplementary Material 1 [file 12884_2024_6580_MOESM1_ESM.docx]
