# Supplementary material for: Preferences of support and barriers and facilitators to help-seeking in pregnant women with severe fear of childbirth in Sweden: a mixed-method study
Source: BMC Pregnancy Childbirth. 2024 May 25;24:388. doi: 10.1186/s12884-024-06580-2 (PMC11127315; doi:10.1186/s12884-024-06580-2)
Supplement: Supplementary file 2 — Supplementary Material 2 [file 12884_2024_6580_MOESM2_ESM.docx]

**APPENDIX II**

**Advertisement for social media.**

*Image*

https://www.facebook.com/forlossningsradslaUU/videos/748514086191221/?__cft__[0]=AZVbYhMJJMKCfE-PFPtcKPMjf8GVoamiSQh-E8L2NV_KUGXPVFdvJ3iL-Mm8d82HIq98yndJjVvxtuA6H3MR4pDt6WjK0_Z8ZzHsa-q-WnDIuscMPqZWZoABWm8B-4U5yV9ZMeowdkYGIrcIvhLqXr4XUPna6oXiwHdhnSgyZAQq8EOZHEQ__rGliYGbgEjy9nV7cE9EkAz4d9WPttSm8MdH&__tn__=-UK-R

*Text*

Är du gravid och känner dig rädd inför förlossningen? Du vet inte hur det ska bli, hur det kommer att kännas. Eller när. Vi vill gärna veta hur du tänker och vårt mål är att ta fram ett stödprogram för blivande föräldrar. Enkäten tar ca 20 minuter att svara på. Stort tack ❤️

https://survey.uu.se/surveys/?s=X4J88L4FDR
