# Supplementary material for: Preferences of support and barriers and facilitators to help-seeking in pregnant women with severe fear of childbirth in Sweden: a mixed-method study
Source: BMC Pregnancy Childbirth. 2024 May 25;24:388. doi: 10.1186/s12884-024-06580-2 (PMC11127315; doi:10.1186/s12884-024-06580-2)
Supplement: Supplementary file 3 — Supplementary Material 3 [file 12884_2024_6580_MOESM3_ESM.docx]

**APPENDIX III**

**Table 1. Background characteristics for pregnant women with severe childbirth fear in relation to parity.**

1. What year were you born?

2004, 2003, 2002, 2001, 2000, 1999, 1998, 1997, 1996, 1995, 1994, 1993, 1992, 1991, 1990, 1989, 1988, 1987, 1986, 1985, 1984, 1983, 1982, 1981, 1980, 1979, 1978, 1977, 1976, 1975, 1974, 1973, 1972, 1971, 1970.

3. What is your current marital status?

- Married/have a partner and are living together
- Married/have a partner, and do not live together
- Single/ divorced/ seperated/ widow

5. If you have a birthing partner, to what extent are you being supported by your partner during this pregnancy?

- To a very large extent
- To a fairly large extent
- To a small extent
- Not at all
- Not relevant

8. Enter your highest level of completed education:

- Primary school
- High school or equivalent
- College/University

7. Have you previously been pregnant? Several options are possible.

- No
- Have given birth to a living child
- My child was stillborn
- I have had a miscarriage
- I have had an abortion

9. What type of employment do you have?

- Employed
- Studying
- Several options are possible. Unemployed
- On sick leave
- Sickness compensation and unable to work
- Parental leave
- Other

11. In what part of the world were you born?

- Sweden
- Other Nordic country (Norway, Denmark, Finland, Iceland)
- Europe
- Africa
- Asia
- Middle East
- North America
- South America
- Oceania
- Other

13. I live in a:

- City
- Town
- Village

16. What week of pregnancy are you in?

- 2
- 3
- 4
- 5
- 6
- 7
- 8
- 9
- 10
- 11
- 12
- 13
- 14
- 15
- 16
- 17
- 18
- 19
- 20
- 21
- 22
- 23
- 24
- 25
- 26
- 27
- 28
- 29
- 30
- 31
- 32
- 33
- 34
- 35
- 36
- 37
- 38
- 39
- 40
- 41
- 42

18. In your opinion, was this pregnancy planned?

- Yes
- Planned but not the exact timing
- No

20. If the pregnancy is normal (not medically

complicated): If you could choose, which method of delivery would

you prefer?

- Vaginal delivery
- Caesarean section
- Do not know

28. Are you experiencing mental health difficulties currently?

- Yes
- No

29. Have you experienced mental health difficulties prior to this pregnancy?

- Yes
- No

30. Have you ever experienced violence in a partnership (e.g. with a partner or spouse)?

- Yes
- No

**Table 2. Supportive treatment preference by pregnant women in relation to severe fear of childbirth and parity.**

38. Have you received any professional treatment for your fear of childbirth?

- Completed treatment
- Ongoing treatment
- Treatment is planned
- No treatment is planned

38. If treatment is completed, on-going or planned, what kind of professional treatment is it/will it be?

- Counselling with a midwife (e g Aurora clinics)
- Counselling with a physician
- Counselling with a psychologist
- Counselling with a councellor/curator
- Cognitive behavioral therapy (CBT)
- Psychodynamic therapy (PDT)
- Psychoeducation (meaning e.g., receiving information about psychological difficulties, for example fear of childbirth, in different formats such as group classes, information leaflets, or online resources)
- Other

38. If no treatment is planned, would you like to receive treatment/support for your fear of childbirth?

- Yes
- No
- I don´t know

40. If treatment is ongoing or completed, to what extent do you think your professional treatment has helped you?

- To a very large extent
- To a large extent
- To a lesser extent
- Not at all

42. How important is talking about your fear of childbirth?

- It is very important
- It is important
- It is not important at all

44. Would you like to receive support during pregnancy, childbirth and parenthood in relation to your experience with fear of childbirth?

- Yes
- No
- I don´t know

46. If you would like to receive professional support for fear of childbirth, whom would you like to deliver the support? Several options are possible.

- Midwife
- Psychologist
- Counsellor
- Physician
- Other professional

47. If you would like to receive support for fear of childbirth, in what form could that support be provided? Several options are possible.

- Individual support for me
- Support for me and my partner
- Group-based support with other mothers
- Group-based support with other mothers and partners

47. If Yes, what types of support would you like to receive for fear of childbirth? Several options are possible.

- Extra visits with an antenatal care midwife during during pregnancy pregnancy
- Parental preparation with antenatal care midwife
- Dialouge with other parents
- Breathing -and relaxation exercises
- Using the method Giving Birth without Fear
- Mindfullness
- Other

**Table 3. Barriers and facilitators in seeking help for pregnant women with severe fear of childbirth.**

39. What obstacles have you experienced in seeking help for your fear of childbirth? Several options are possible.

- Stigma (unwanted social stigma) surrounding fear of childbirth
- Previous negative experience of healthcare contacts
- Fear of not being believed in by others
- Fear of not being listened to by others
- Discomfort of having to face my own fears
- Other

40. What would make it easier to seek help for fear of childbirth? Several options are possible.

- Receiving an appointment time as soon as I tell a professional about my fear of childbirth
- Receiving professional support close to my home
- Being able to choose between different times for appointments
- Being able to receive professional support outside of standard working hours
- Other

45. If you would like to receive support for fear of childbirth, how would that support look like? Several options are possible.

- Physical meetings with midwife, curator, psychologist or physician
- Online meetings (e.g., video-conference)
- Telephone support with midwife, curator, psychologist or physician
- Text messages (SMS)
- Chat forums (e.g., Familjeliv)
- Internet link to e.g. information, illustrations, films, tips on literature
- Printed brochure with e.g. information, illustrations, references to movies, tips on literature
- Mobile application
- Other
